# Miniaturized Dual-Band Bandpass Filter Based on High-Order Mode of Spoof Surface Plasmon Polaritons with Triangular Notch Structure

**DOI:** 10.3390/s26206430

**Published:** 2026-10-12

**Authors:** Yan Liu, Huarui Zhang, Shige Wei, Cong Chen, Hao Luo, Ke Gong

**Affiliations:** College of Physics and Electronic Engineering, Xinyang Normal University, Xinyang 464000, China; liuyan6825@163.com (Y.L.); 17828597984@163.com (H.Z.); weishige2000@163.com (S.W.); easterncabbage@163.com (C.C.); lhxytc@163.com (H.L.)

**Keywords:** spoof surface plasmon polaritons (SSPPs), dual-band bandpass filter, triangular notch, high-order mode

## Abstract

This paper proposes a novel triangularly notched spoof surface plasmon polariton (SSPP) unit for designing dual-band bandpass filters (DPF). Compared with rectangular SSPP units, the proposed triangularly notched SSPP unit exhibits a superior slow-wave effect and enables the simultaneous excitation of multiple higher-order SSPP modes through a simple structure with compact transverse and longitudinal dimensions. The designed filter optimizes the need for conventional mode-conversion transition structures, significantly reducing the overall size. By adjusting the geometric parameters of the proposed triangularly notched SSPP unit, the upper and lower cutoff frequencies of the passbands can be flexibly tailored with fewer adjusting parameters. The final filter achieves two operating passbands covering 4.85 GHz to 9.3 GHz and 10.4 GHz to 15.3 GHz, with the return loss maintained better than 12 dB within both bands. For validation, the designed filter was fabricated and measured. The measured results show good agreement with the simulations, verifying the effectiveness of the proposed structure and design methodology.

## 1. Introduction

With the continuous advancement of modern wireless communication technologies and the rapid growth of communication equipment, there is an increasing demand for better interference resistance. To enhance the anti-interference capability of the equipment, dual-band filters [1,2,3] demonstrate significant potential in communication systems. Multi-mode resonance technique has been employed in the design of a dual-band filter, while some works [4,5,6,7] conducted on substrate integrated waveguide (SIW) platforms have demonstrated excellent performance. Recently, dual-band filter based on spoof surface plasmon polariton (SSPP) has also garnered widespread attention.

Spoof surface plasmon polaritons (SSPPs) are artificial electromagnetic modes that are achieved by constructing sub-wavelength periodic structures (such as arrays of holes, slits, or corrugated stripes) on metal surfaces [8]. This allows SSPP to mimic the behavior of natural surface plasmon polaritons (SPPs) in the microwave and terahertz frequency ranges. SPPs are electromagnetic wave modes that exist at the interface between metals and dielectric materials, possessing the ability to propagate beyond the diffraction limit and exhibiting significant localized field enhancement effects. However, at lower frequencies, such as microwaves, metals behave as perfect electric conductors (PEC), making it difficult for electromagnetic waves to penetrate the surface, which prevents the natural existence of such surface waves. To overcome this limitation, Pendry proposed in 2004 the design of periodically patterned grooves or hole arrays on metal surfaces, thereby extending the unique properties of SPPs to the terahertz and microwave bands [9]. The artificially guided surface waves formed in this manner are referred to as SSPP.

Spoof surface plasmon polaritons as a special type of artificial periodic structure, can simulate the propagation of natural surface plasmon polaritons at the metal-dielectric interface, exhibiting exponential decay in the terahertz and even lower frequency ranges [10,11,12,13]. SSPP features highly tunable dispersion characteristics, allowing flexible control of its propagation behavior by adjusting the geometric parameters of the unit cells. As a result, it demonstrates significant application potential in various microwave and terahertz devices, such as power dividers [14,15], transmission lines (TL) [16,17,18,19], filters [20,21], antennas [22,23,24,25], and polarization converters [26], thus providing new possibilities for the development of technology in this field. In 2005, Hibbins and colleagues experimentally observed SSPP modes with tunable cutoff frequencies [27]. Following this, the field attracted considerable research attention, offering unprecedented new avenues for electromagnetic wave manipulation. To address the complexity associated with earlier three-dimensional structures, researchers proposed planar SSPP structures, which also exhibit characteristics such as efficient transmission, strong field confinement, and weak coupling. Consequently, these planar structures have found widespread applications in microwave circuits and systems [28,29,30].

It is noteworthy that traditional feeding methods have limitations in directly exciting SSPP waves, typically requiring specific transition structures to achieve effective conversion between the conventional transmission line mode and the SSPP mode. This conversion needs to account for issues related to impedance matching and mode matching, which can lead to an overall increase in size and additional signal loss—an aspect that is particularly critical in high-frequency and high-bandwidth applications. Previous research has mainly focused on the fundamental mode (mode 0) of SSPP, while studies on its higher-order modes have been relatively scarce. Unlike the low-pass characteristics exhibited by the fundamental mode, the higher-order modes of SSPP display a natural band-pass characteristic, enabling them to form excellent transmission performance passbands under appropriate excitation conditions [31]. Consequently, exploring the higher-order modes of SSPP not only contributes to understanding their propagation characteristics and physical mechanisms but also holds significant practical implications for the design and optimization of novel band-pass filters. The introduction of higher-order modes provides more degrees of freedom in filter design, allowing effective selection and suppression of signals across different frequency ranges, thereby optimizing system performance and reliability. Furthermore, studying the coupling characteristics, resonant behavior, and interactions with other modes of SSPP’s higher-order modes can assist in developing new waveguide structures, further advancing fields such as nanophotonics, wireless communication, and radio-frequency devices.

In the published literature, the concept of designing dual-band bandpass filters using higher-order modes of SSPP involves first designing a wideband bandpass filter and then introducing suppression within the passband [32,33,34,35]. However, this method often requires additional resonant structures. The meandered-line SSPP unit can simultaneously excite multiple higher-order modes of SSPP, and its potential application in multiband bandpass filters has already been demonstrated [36,37,38]. The Twin-Arrow [39] and Tree-Branch [40] SSPP structure are also the classic approach for designing dual-band filters. However, when multiple higher-order modes coexist, precisely manipulating a single higher-order mode becomes extremely challenging due to the complex interactions and coupling relationships between the various higher-order modes. Therefore, in the design of multiband bandpass filters utilizing multiple higher-order modes of SSPP, both the passband of a single higher-order mode and the stopbands between different higher-order modes are fixed. Although the aforementioned studies have successfully employed higher-order modes of SSPP to achieve specific filtering functions, further optimization and improvements are still necessary to better meet the growing demands of modern communication systems.

This article focuses on the optimization of traditional rectangular SSPP cell structures and proposes a novel triangularly notched SSPP unit. This new cell achieves the design of a dual-band bandpass filter by trimming a portion of the triangular shape from the top and bottom of the traditional structure, thereby promoting the excitation of higher-order modes. The proposed triangularly notched SSPP unit exhibits significant advantages: First, this structure substantially enhances the level of integration, improving the transitional structures required for traditional feeding approaches, thereby overcoming the limitations of rectangular SSPP unit in terms of longitudinal dimensions. This effectively excites multiple higher-order modes and considerably minimizes the longitudinal size of the unit. Secondly, the notch-type structure improves the selectivity of the higher-order modes. By adjusting the height and period of the unit, the second and third higher-order mode can be effectively placed within the stopbands of the first and the fourth higher-order modes. By combining the first and fourth higher-order patterns, a dual-band bandpass filter with the desired performance can be constructed. This innovative design not only optimizes the structural dimensions but also provides new ideas and methods for achieving efficient and flexible frequency selectivity.

## 2. Analysis of Cell Structure

**Figure 1:**
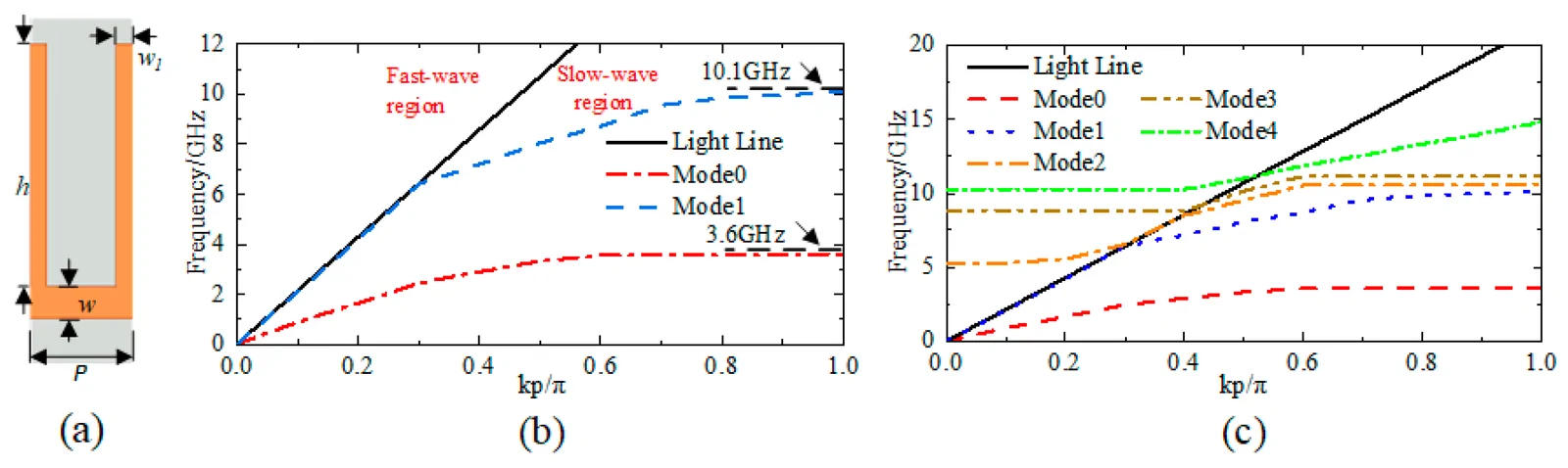
(**a**) Schematic diagram of a conventional rectangular unit cell structure. (**b**,**c**) its dispersion curves.

The schematic diagram of a conventional rectangular SSPP unit is shown in Figure 1a. The unit is designed on a Rogers RT5880 substrate with a relative permittivity (*ε_r_*) of 2.2 and a thickness of 0.508 mm. To enhance the confinement of the electromagnetic field, a metal ground plane is implemented on the bottom side of the substrate, which significantly simplifies the design of the transition structure required for exciting SSPPs from a microstrip line and facilitates the mode conversion from quasi-TEM to SSPP modes. For the unit, the geometric parameters h and p are set to 16 mm and 7 mm, respectively, meeting the excitation condition for higher-order modes of the SSPP structure [41]. To clearly observe and study the dispersion characteristics of the SSPP unit, eigenmode analysis is performed using electromagnetic simulation software (CST Microwave Studio 2021). By comparing with the propagation of light in free space, the dispersion curves corresponding to the rectangular SSPP unit with a longitudinal height h of 16 mm are plotted, revealing the behavior of mode 0 and mode 1.

As shown in Figure 1b, the dispersion curve lies to the right of the light line, confirming the slow-wave behavior of the SSPP mode during propagation. Unlike the fundamental mode (mode 0) of the SSPP unit, which exhibits low-pass characteristics, the first higher-order mode (mode 1) inherently demonstrates bandpass response, with a lower cutoff frequency of 6.4 GHz and an upper cutoff frequency of 10.1 GHz. Furthermore, the dispersion curves indicate that the fundamental mode deviates more significantly from the light line compared with the higher-order modes. Therefore, when constructing a bandpass filter by integrating the fundamental mode with other high-pass structures, it is essential to incorporate an SSPP transition section capable of achieving both wavevector and impedance matching. In contrast, the higher-order modes of the SSPP unit can be directly utilized to form a passband with favorable transmission performance.

**Figure 2:**
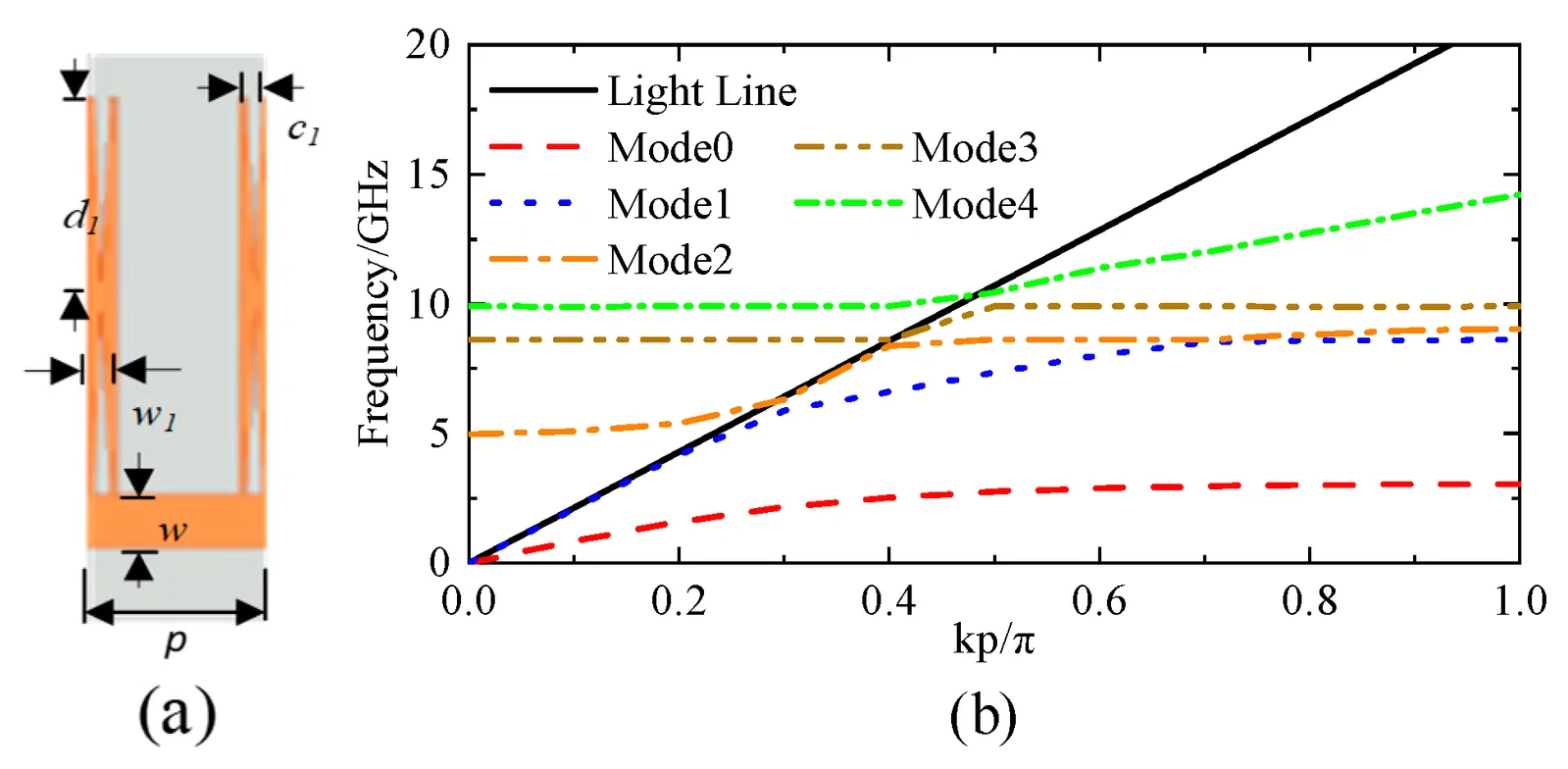
(**a**) Schematic diagram of the triangular notch type unit structure. (**b**) Its dispersion curves.

Furthermore, as shown in Figure 1c, the conventional rectangular unit cell structure has the ability to excite the fundamental mode and more higher-order modes under appropriate excitation conditions. It is obvious that modes 2 and 3 are close to each other, which makes it quite difficult to excite them both at the same time. The bandwidth of mode 2 or mode 3 is narrow and their operating bands are between the upper cutoff frequency of mode 1 and the lower cutoff frequency of mode 4. Employing the cutoff characteristics of an operating frequency band to suppress unwanted modes represents a viable suppression technique [42]. If the dual-band bandpass filter is operating on mode 1 and mode 4, the impact of mode 2 and mode 3 can be better depressed, because they happen to fall within the stopband range associated with modes 1 and 4. Although improved stopband performance is desirable, the longitudinal dimensions and limited tunable parameters of the rectangular SSPP unit greatly constrain its applicability in filter miniaturization and higher-order mode regulation [12]. Through the eigenmode analysis, the conventional rectangular unit cell structure can excite fundamental mode and more higher-order modes as shown in Figure 1c. Obviously, this needs the appropriate excitation conditions, such as the impedance matching condition, inter-band rejection, out-of-band rejection and so on. The ideal design is to excite the higher-order modes without appending new coupling structure while maintaining the overall dimension of the unit unchanged. To address this issue while simultaneously exciting two higher-order modes with enhanced stopband performance, a novel triangularly notched SSPP unit is proposed, as illustrated in Figure 2a. This is good for the miniaturized design. Without altering the original configuration, two triangular notches are etched on the upper and lower edges of the rectangular SSPP unit. This modified geometry increases the current path length and the relative effective electrical length of the SSPP waveguide, thereby providing stronger field confinement and improved dispersion control. Figure 2 depicts the structural layout and dispersion characteristics of the triangularly notched SSPP unit.

The results indicate that, within the same period, the triangular-notch SSPP unit exhibits the following characteristics: Mode 0 has a low asymptotic frequency of 3 GHz; Mode 1 has a starting frequency of 4.4 GHz and an asymptotic frequency of 8.6 GHz; Mode 4 has a starting frequency of 10 GHz and an asymptotic frequency of 14.2 GHz. In contrast to the dispersion curves of the conventional rectangular unit, this design not only lowers the cutoff frequencies of modes 0 and 1 but also improves the stopband performance. First, the dispersion curve of mode 0 becomes smoother and the upper cutoff frequency is away from the lower cutoff frequency of mode 1, which is beneficial to depress the impact of mode 0 with appropriate feeding structure. Second, the curves of modes 2 and 3 are also smoother and their locations can be moved. It means that modes 2 and 3 can be installed on an appropriate location to reduce the influence on modes 1 and 4. Furthermore, by examining the deviation of the two modes from the light line, it can be concluded that for mode 1 of the proposed triangular-notch SSPP unit, a passband can be formed with requiring an simpler SSPP transition structure contrast to the conventional rectangular unit; whereas designing a passband using mode 0 necessitates an SSPP transition structure to achieve wave vector and impedance matching. Thus, based on the miniaturized design, modes 1 and 4 are mainly excited and used to design the dual-band filter. Since mode 0 is relatively far from mode 1, the influence of mode 0 can be better depressed by the cut-off effect of the lower stopband of mode 1 [42].

**Figure 3:**
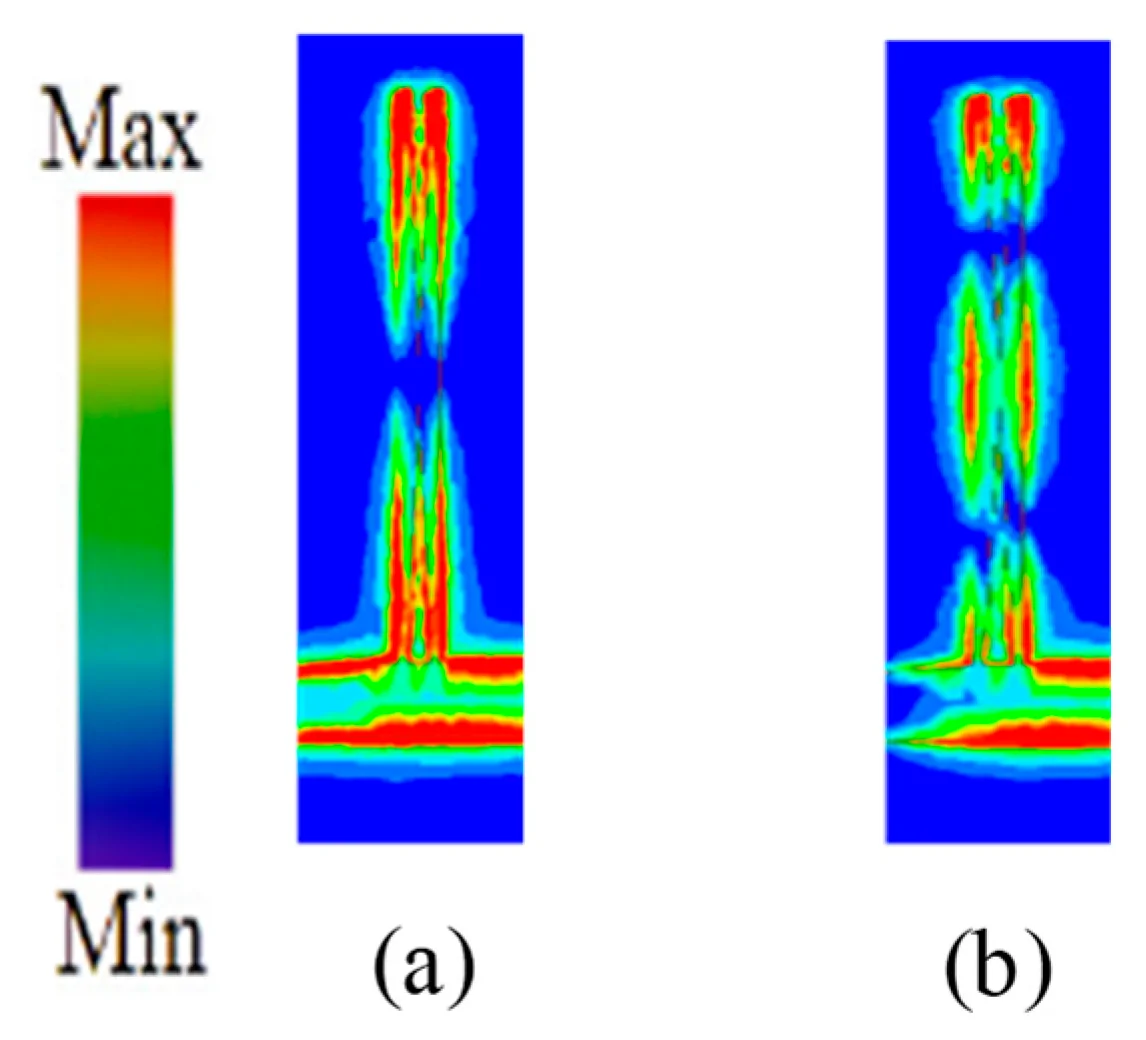
The electric field distribution of the dispersion unit at (**a**) 7 GHz and (**b**) 13.5 GHz.

To further clarify the mechanism of mode controllability for mode 1 and mode 4 of the proposed triangularly notched SSPP unit, simulated electric field distributions at each corresponding operating frequency within their working bands are presented in Figure 3. Distinct modal patterns can be observed in the electric field distributions of these two modes. Specifically, the electric field of mode 1 is primarily concentrated in the upper and lower regions of the SSPP unit. In contrast, the electric field of mode 4 is distributed across the upper, middle, and lower regions of the SSPP unit.

**Figure 4:**
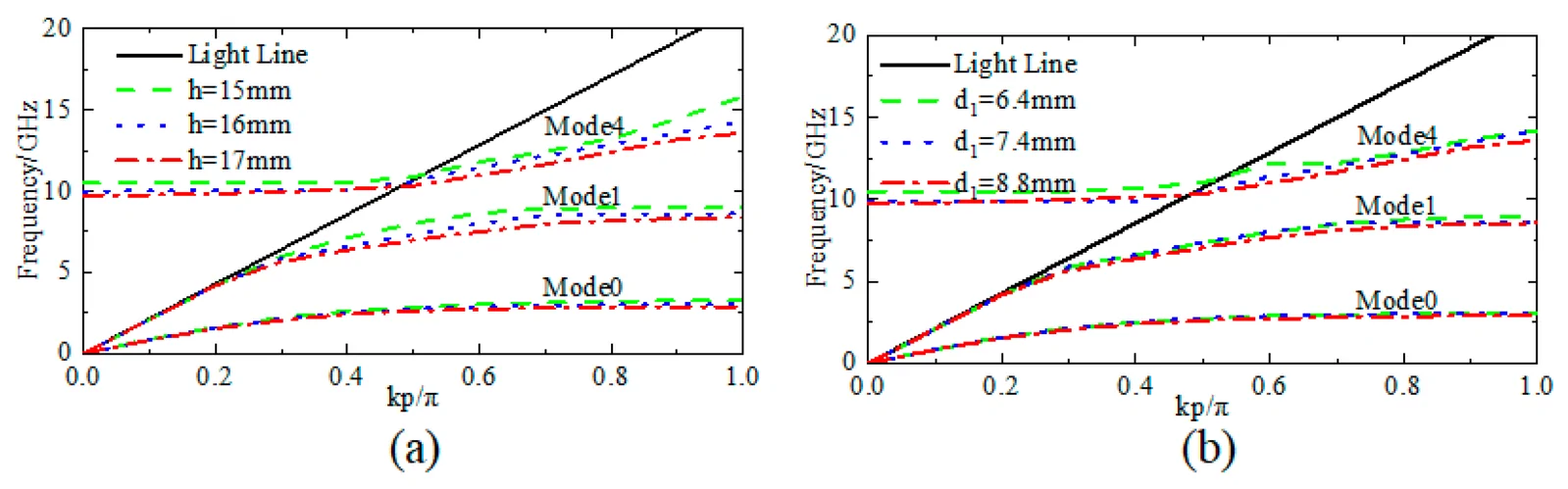
(**a**) Dispersion curves of the triangular notch SSPPs unit with different *h* from 15 to 17 mm. (**b**) Dispersion curves of the triangular notch SSPPs unit with different *d*_1_ from 6.4 to 8.8 mm.

After completing the analysis of the electric field distribution of the triangular notch SSPPs unit, further investigation was conducted on the effect of its geometric parameters on dispersion characteristics, focusing primarily on the variation patterns of mode 1 and mode 4. As shown in Figure 4a, by adjusting the unit height h and the triangular notch length d_1_, the passband characteristic can be controlled and exhibits good consistency. This is good for the miniaturized design. Even if the unit height h is set to a fixed value, the two passbands can still be adjusted through optimizing the triangular notch structure. When the unit height h increases from 15 mm to 17 mm, the asymptotic frequency of mode 1 remains relatively unchanged; meanwhile, the cutoff frequency of mode 1 decreases from 9 GHz to 8.4 GHz, while the starting frequency and cutoff frequency of mode 4 decrease from 10.4 GHz and 14.8 GHz to 9.8 GHz and 14 GHz, respectively. The results in Figure 4b indicate that the variation in the triangular notch height d_1_ has little impact on the cutoff frequency of the fundamental mode; however, both the starting frequency and cutoff frequency of mode 1 and mode 4 decrease as d_1_ increases, albeit the degree of change is less pronounced compared with the effect of the unit height h. In other words, the dispersion curves with different parameter d_1_ exhibit better consistency. The proposed triangular notch structure possesses the potential and capability to further optimize the passband performance.

**Figure 5:**
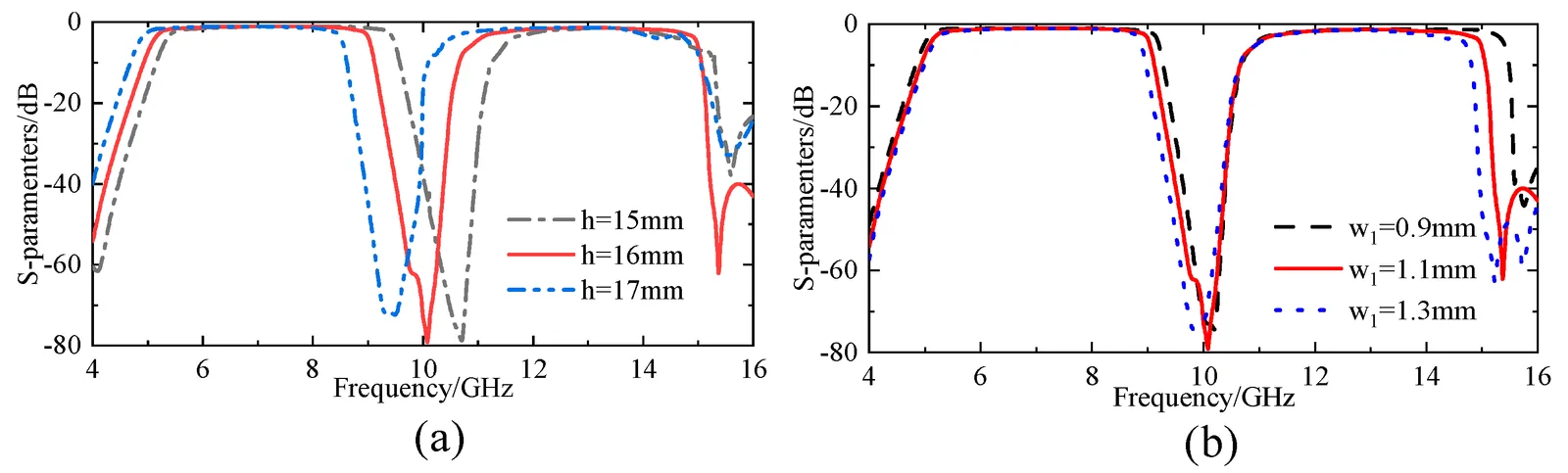
(**a**) Simulated transmission coefficient (S_21_) of the designed BPF with *h* parameters. (**b**) Simulated transmission coefficient (S_21_) of the designed BPF with *w*_1_ parameters.

It is obvious that the triangular notch parameter d_1_ is closely related to SSPP unit geometric parameters h. Thus, in order to balance the passband performance and the SSPP unit size, the geometric parameters h and w_1_ are used to investigate the effects on the proposed structure. When the units are integrated together, altering the parameter h is mainly to affect the cutoff band of the dual-band filter as shown in Figure 5a, the upper and lower cutoff frequencies of the passband decrease significantly as h increasing, which are consistent with the change in the dispersion curve in Figure 4a. Meanwhile, the change in the lower cutoff frequency of the first passband is slightly more pronounced than the change in the upper cutoff frequency of the second passband. Also, under the fixed parameter h, adjusting the width parameter w_1_, the upper cutoff frequency of the second passband can be controlled as shown in Figure 5b. And the stopband of the filter can also be optimized by the parameter w_1_. Based on these studies, the triangular notch structure will play an important role in the miniaturized design.

The geometric parameters of the proposed triangular notch SSPPs unit exhibit a high degree of consistency with the patterns observed in the electric field distribution and their influence on dispersion characteristics. Leveraging this relationship, precise and appropriate adjustments to the geometric parameters of the triangular notch SSPP structure enable the customized design of its desired dispersion properties. Notably, the upper and lower cutoff frequencies of the passbands generated by mode 1 and mode 4 are adjustable within this configuration. A thorough analysis of the dispersion curves provides critical insights that can serve as a foundational reference for the design of a dual-band bandpass filter, with the first passband set to operate within the frequency range of 4.4 to 8.6 GHz and the second passband ranging from 10 to 15.6 GHz.

## 3. Filter Structure Based on DFP SSPP

**Figure 6:**
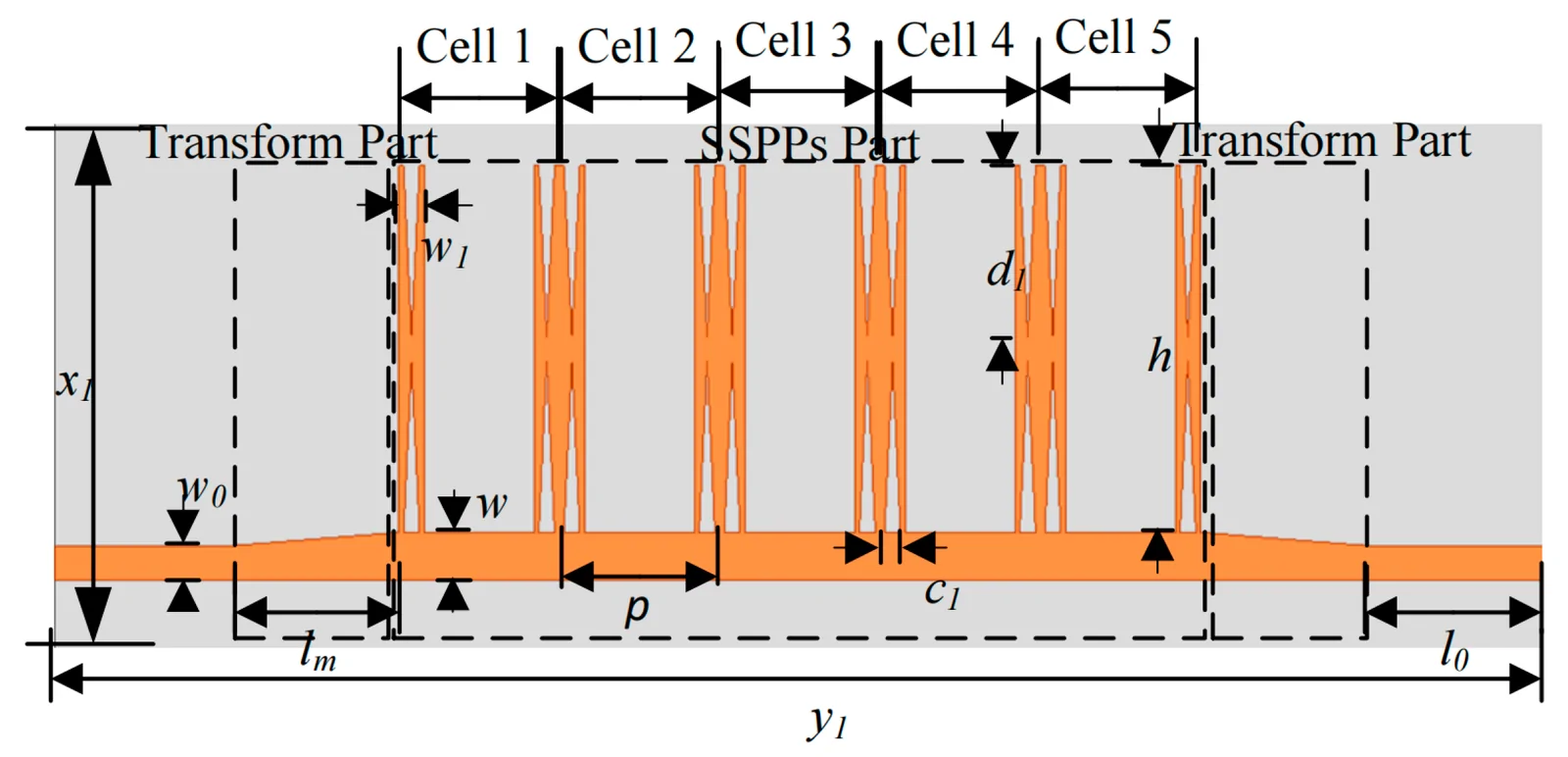
The schematic diagram of the designed DPF based on the proposed triangular notch SSPPs unit.

Based on the proposed triangular-notch SSPP unit, a compact dual-band bandpass filter has been designed, as illustrated in Figure 6. The filter structure consists of two main parts. The first part is a microstrip transmission line with a width of 1.5 mm and a length of 7.5 mm, providing 50 Ω impedance matching. The second part is the SSPP transmission section, which is composed of five SSPP units labeled in Figure 6. Unlike most existing SSPP-based filters—which typically include a transmission line, a transition section for coupling to the SSPP structure, and the SSPP transmission section—the proposed design is directly connected to the SSPP units via a wedge-shaped microstrip impedance matching line. This leads to a more compact layout. The filter directly utilizes the bandpass characteristics of mode 1 and mode 4 of the SSPP units, allowing excitation via a simple microstrip feed and further aiding miniaturization. All parameters of the proposed structure are summarized in Table 1. Although the SSPP transition section is simplified, the design effectively suppresses the low-pass effect associated with mode 0, while achieving excellent dual-band passband performance driven by modes 1 and 4.

**Figure 7:**
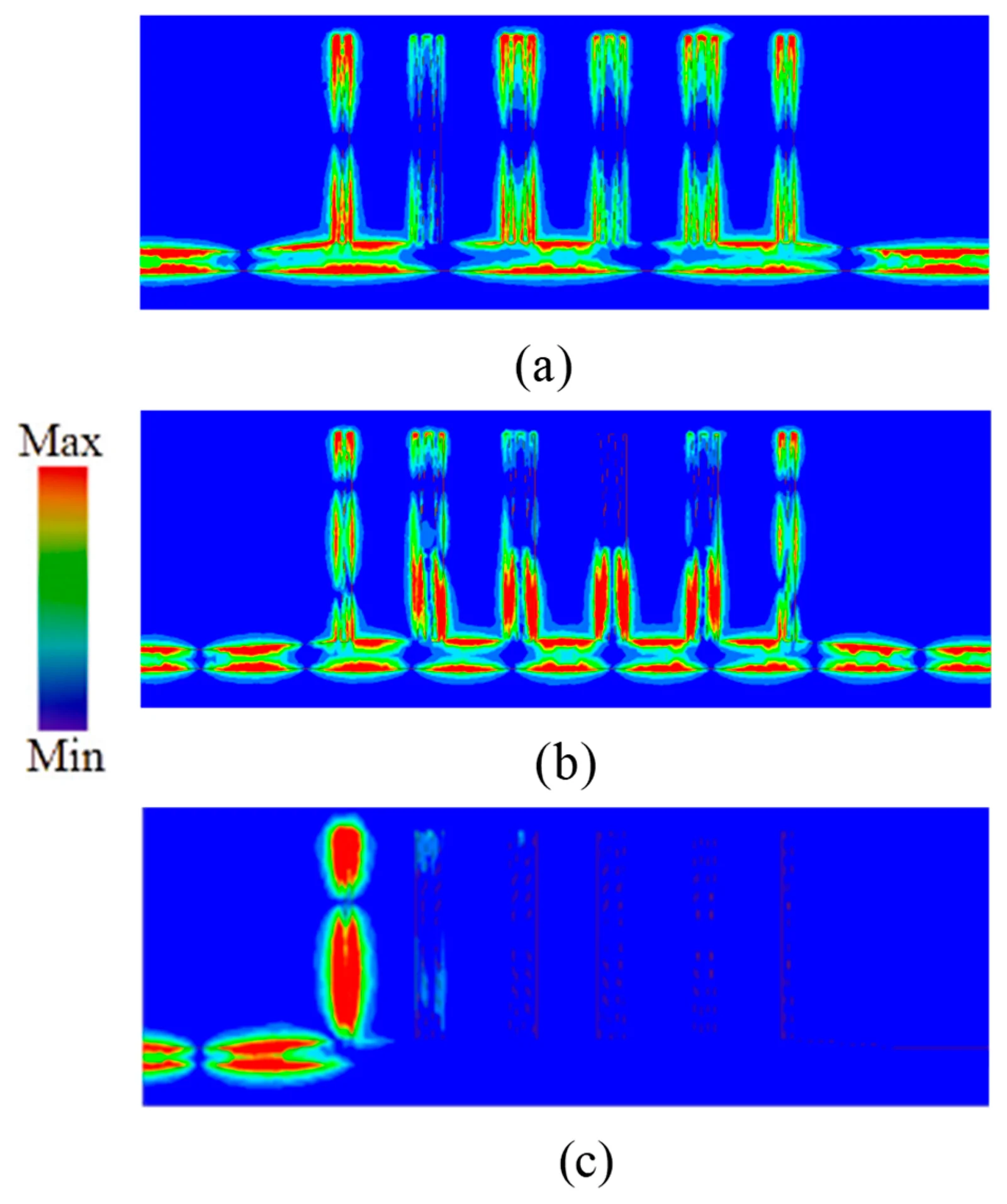
(**a**) Electric field distribution at 7 GHz. (**b**) Electric field distribution at 13.5 GHz. (**c**) Electric field distribution of stopband.

To further investigate the electromagnetic characteristics of the SSPP device, the systematic measurement of the overall electric field distribution of its structure is conducted. Figure 7 visually presents the electric field distributions for mode 1 and mode 4. From the observations of the electric field distribution diagrams, it can be noted that mode 1 has its electric field primarily concentrated on the upper and lower sides of the SSPP unit, while the electric field of mode 4 is distributed across the upper and lower sides as well as the center of the SSPP unit. Furthermore, when examining the electric field at the stopband frequency of 10 GHz, it was found that the electromagnetic waves were effectively suppressed and could not penetrate through this structure. This result confirms the rationality and effectiveness of the proposed structure design.

## 4. Experimental Verification of the Filter

**Figure 8:**
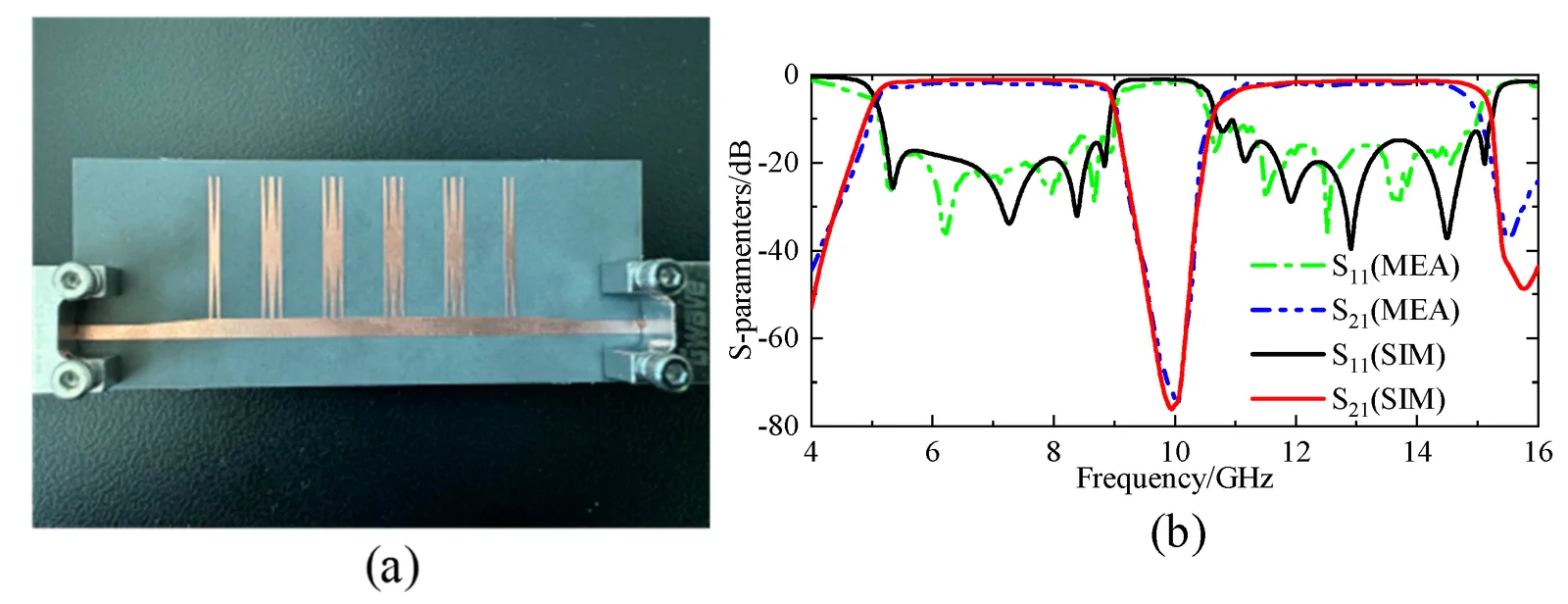
(**a**) Fabricated prototype of the Dual-bandpass filter. (**b**) Comparison of measured (MEA) and simulated S-parameters.

To validate the simulation results of the triangular notch SSPP dual-band bandpass filter, a prototype was fabricated using traditional PCB technology on a 0.508 mm thick Rogers RT5880 substrate. Figure 8a illustrates the physical realization of the dual-band bandpass filter, where the yellow section represents the copper metallization layer and the black section denotes the Rogers 5880 dielectric substrate.

At the end of the microstrip line, two standard SMA connectors were connected, and the S-parameters were characterized using a vector network analyzer (Keysight N5222A). According to the measured results shown in Figure 8b, the frequency range of the first passband is from 4.85 to 9.3 GHz, and the second passband range is from 10.4 to 15.3 GHz, with the insertion loss better than 1.7 dB and the return loss better than 12 dB. The measured results are in good agreement with the simulated data, validating the proposed design concept and the effectiveness of the structure. The specific simulated and measured data are presented in Table 2.

The slight discrepancies between the simulated and measured results are present as shown in Figure 8b. In the second passband, the return loss gradually increases as the band edge is approached, thus increasing the insertion loss. Compared with the first passband, this phenomenon is more pronounced in the second passband. First, to achieve a miniaturized design, a simple triangular notch structural unit is adopted to realize a two-passband design. The unit structure is relatively simple and has limited control parameters. Compared with complex structures, the tuning capability has certain limitations. Secondly, this phenomenon is more pronounced in the upper sideband of the first passband and the lower sideband of the second passband, primarily due to the higher-order mode 2 and mode 3. This corresponds to the dispersion curve characteristic shown in Figure 2b. On the one hand, it needs to make the mode 2 and mode 3 dispersion curves smoother and closer to each other, which is beneficial to eliminate their influence. On the other hand, it is better to expand the passband bandwidth and constrain the mode 2 and mode 3 dispersion curves far from the two passbands. The final structure parameters listed in Table 1 are determined by comprehensive consideration. The proposed structure may not be the optimal choice for suppressing modes 2 and 3. However, given the simple unit structure and miniaturized design, this dual-band bandpass filter still exhibits excellent bandpass performance, and the simulated and measured results are in good agreement. Future work will focus on suppressing higher-order modes to further enhance the filter performance. Meanwhile, the upper cutoff frequency of the second passband is decreased compared with the simulated result. As shown in Figure 5b, the upper cutoff frequency of the second passband decreases as the branch width w_1_ increases. This may be mainly attributed to manufacturing tolerances and non-ideal factors in the measurements.

In order to validate and reproduce this work better, the related simulated and measured data are also presented.

For the simulations: (i) the eigenmode analysis was carried out with periodic boundary conditions along the *X*-axis and open boundary conditions along the *Y*- and *Z*-axes; (ii) the unit cell was modeled with periodic boundary conditions, and the phase difference was swept from 0° to 180° along the X direction; (iii) the structure was excited through a waveguide port with a reference impedance of 50 Ω; (iv) the conductors were modeled as copper with a thickness of 35 μm and a conductivity of σ = 5.8 × 10^7^ S/m, with conductor losses included; (v) the substrate was described by a relative permittivity of *ε_r_* = 2.2 and a loss tangent of tan δ = 0.0009 at 10 GHz; (vi) an adaptive meshing scheme was used; and (vii) the simulations were considered converged when the S-parameters changed by less than 0.1% between successive mesh refinements.

For the measurements: (i) the S-parameters were measured with a Keysight N5222A network analyzer; (ii) a full two-port SOLT (short–open–load–thru) calibration with isolation was performed over the 0.01–26.5 GHz band using a Keysight 85052D 3.5 mm mechanical calibration kit; (iii) the calibration reference planes were set at the SMA connector planes; (iv) the frequency sweep covered 4–16 GHz with 1601 points and an IF bandwidth of 1 kHz; and (v) the SMA connectors were soldered directly onto the PCB, and their residual effects were removed by a two-port thru de-embedding.

Table 3 presents a measured performance comparison between the proposed triangular notch SSPP filter and those previously reported with the high-order mode. Compared with the traditional SSPP structure, the designed filter demonstrates certain advantages in terms of IL, RL, relative bandwidth, and horizontal size.

## 5. Conclusions

This paper presents a compact dual-band bandpass filter based on triangular notch SSPP units. By tuning the relevant parameters of the triangular notch SSPP unit, the lower and upper cutoff frequencies of the filter can be effectively customized. In addition, the etched notch significantly enhances the stopband performance between the two passbands. The simulation results of the fabricated prototype closely match the actual measurements, thereby validating the effectiveness of the design. This study presents notable advantages, including a compact structure, wide bandwidth, low insertion loss, and dual-band operation, which can significantly facilitate the advancement of compact, multifunctional microwave systems leveraging SSPP structures.

## Figures and Tables

**Table 1:** Parameters of the designed DPF (unit: mm).

Parameters	Size	Parameters	Size	Parameters	Size
x_1_	23	w_1_	1.1	d_1_	7.4
y_1_	65	w	2.2	h	16
l_0_	7.5	p	7	w_0_	1.5
l_m_	7.5	c_1_	0.7		

**Table 2:** Simulated and measured data of the proposed DPF.

Obj.	Cutoff Freq. (GHz)	IL (dB)	RL (dB)	f_0_ (GHz)	FBW (%)
Sim.	4.4–8.6, 10–15.6	1, 1.5	18, 12	6.5, 12.8	65, 44
Mea.	4.85–9.3, 10.4–15.3	1.5, 1.7	17, 12	7.07, 12.85	63, 38

**Table 3:** Comparison of the proposed DPF with the previous work.

Ref.	Periodic Size (*λ*_0_)	RL (dB)	IL (dB)	f_0_ (GHz)	FBW (%)
[6] *	0.84 × 1.31	17/20	2.02/1.82	5, 8.5	6.26, 7.75
[7] *	0.95 × 1.22	22/15	1.16/2.32	12, 17	6.87, 3.26
[33]	0.59 × 0.9	10/10	2/4	5.2, 7.6	38,17
[36]	0.15 × 0.27	14/10	0.7/1	5.4, 9.7	67, 37
[37]	0.21 × 0.35	13	3	8	49.8
[38]	0.2 × 0.36	10/8	0.6/0.7	7.95, 14.65	-, 29.4
This Work	0.16 × 0.3	17/12	1.5/1.7	7.07, 12.85	63, 38

* SIW Technology.

## Data Availability

The original contributions presented in this study are included in the article. Further inquiries can be directed to the corresponding author.

## Abbreviations

The following abbreviations are used in this manuscript:

SSPP	Spoof surface plasmon polariton
SPP	Surface plasmon polariton
DPF	Dual-band bandpass filter
PEC	Perfect electric conductor
TL	Transmission lines
PCB	Printed Circuit Board
